# Characterising the extent and nature of digital food and beverage marketing in Singapore: a descriptive study

**DOI:** 10.1017/S1368980024002428

**Published:** 2024-12-12

**Authors:** Xin Hui Chua, Clare Whitton, Stefanie Vandevijvere, Bridget Kelly, Rob M van Dam, Salome A Rebello

**Affiliations:** 1 Saw Swee Hock School of Public Health, National University of Singapore and National University Health System, Singapore, 117549 Singapore; 2 School of Medical and Health Sciences, Edith Cowan University, 270 Joondalup Drive, Joondalup, WA 6027, Australia; 3 Department of Epidemiology and Public Health, Sciensano (Scientific Institute of Public Health), Brussels, Belgium; 4 Early Start, School of Health and Society, University of Wollongong, Northfields Avenue, Wollongong, NSW 2522, Australia; 5 Department of Exercise and Nutrition Sciences, Milken Institute School of Public Health, The George Washington University, Washington, DC 20037, USA

**Keywords:** Advertisements, Marketing, Online, Food and beverage, Social media, Asian

## Abstract

**Objective:**

*To characterise the nature of digital food and beverage advertising in Singapore.*

**Setting:**

Food and beverage advertisements within twenty clicks on the top twelve non-food websites and all posts on the Facebook and Instagram pages of fifteen major food companies in Singapore were sampled from 1 January to 30 June 2018.

**Design:**

Advertised foods were classified as being core (healthier), non-core or mixed dishes (e.g. burger) using the WHO nutrient profile model and national guidelines. Marketing techniques were assessed using published coding frameworks.

**Participants:**

NA

**Results:**

Advertisements (*n* 117) on the twelve non-food websites were largely presented as editorial content. Food companies posted twice weekly on average on social media sites (*n* 1261), with eatery chains posting most frequently and generating the largest amount of likes and shares. Key marketing techniques emphasised non-health attributes, for example, hedonic or convenience attributes (85 % of advertisements). Only a minority of foods and beverages advertised were core foods (non-food website: 16·2 %; social media: 13·5 %).

**Conclusions:**

Top food and beverage companies in Singapore actively use social media as a platform for promotion with a complex array of marketing techniques. A vast majority of these posts were unhealthy highlighting an urgent need to consider regulating digital food and beverage advertising in Singapore.

Overweight and obesity prevalence is increasing rapidly in Singapore^(1)^, a city-state in Southeast Asia and worldwide^(2)^. In 2020, 39·3 % of adult Singaporeans were overweight, of which 10·5 % were obese^(1)^. Globally, the food marketing environment has been identified as one of the main drivers of the obesity epidemic^(3)^. In children, exposure to unhealthy food advertising, which promotes food high in saturated fats, trans-fatty acids, free sugars and/or sodium, is associated with a subsequent increase in the consumption of unhealthy foods and influences the development of norms about these foods^(4)^. Fewer studies exist in adult populations as they are considered less vulnerable to advertising, as compared to children^(5)^. However young adulthood is the life stage associated with the most weight gain, both in Singapore^(6)^ and in western settings^(7)^. A recent review indicated that in young adults, aged 18–30 years, exposure to digital marketing of unhealthy commodities was associated with greater use and more positive beliefs about these foods^(8)^. The WHO has defined digital marketing as ‘promotional activity, delivered through a digital medium, that seeks to maximize impact through creative and/or analytical methods’^(9)^. This targeted approach is likely to be more effective than traditional static marketing methods. Marketers are allocating more budget to digital advertising, with a continuing and consistent move from offline media to digital advertising using persuasive strategies, in line with time-use trends in which consumers are spending increasing amounts of time online. In a 2019 survey of forty-five economies, time spent online ranged from 6·2 to 9·1 h/d^(10)^. Singapore adolescents and adults fall above the middle of this range spending 8·1 h/d using the internet^(11)^. Globally around one-third of the time spent online is on social media^(12)^. In Singapore, 84·4 % of adults and adolescents are active social media users, and this group spends over 2 h/d on social media^(11)^. Internet, and in particular social media, marketing allows for a level of personalisation that can include not only demographics but also considers past behaviours and caters to individuals’ interests.

Recently, the WHO called for the mapping of global, regional and national digital marketing ecosystems and also noted the failure of member states to fully implement its recommendations on food and beverage marketing and the ineffectiveness of voluntary schemes towards reducing the exposure of digital marketing among children^(13)^. For example, in the European region^(14)^, many countries have taken no action or have implemented policies or regulations employing very narrow criteria, with less than a third of countries including marketing on the internet^(15)^. Current regulations on unhealthy food marketing across the Western Pacific region are largely voluntary, thus reinforcing the importance of monitoring in the region^(15)^. In Singapore, guidelines to prevent unhealthy food marketing to children are established, but this is limited in scope and is self-regulated^(16)^. Comprehensive monitoring of digital marketing activities can evaluate the impact and identify potential entry points to strengthen and develop better regulatory guidelines^(13,17)^.

Only a few studies, primarily from Australia and New Zealand, have characterised digital food and beverage marketing techniques^(18–20)^, with most focusing on a single media platform (e.g. Facebook). Monitoring multiple digital platforms is important to gain a comprehensive understanding of food industry marketing techniques for the implementation of evidence-based policies towards restricting food marketing exposure among children^(5,8)^. There is also a lack of studies in Asian countries, in which differences in regulations, food preferences, dietary behaviours and socio-cultural factors may attract an alternate suite of marketing techniques as compared with Western populations^(17)^.

We aimed to conduct a scan of digital environments to characterise the nature of food and beverage marketing in Singapore – including the types of foods advertised and the key marketing techniques of major food companies. We also sought to explore two top digital social media platforms, Facebook and Instagram, to understand the volume of marketing activity in this space. We focused on websites that were not owned by the food industry, where people are more likely to be incidentally exposed to food and beverage advertising, to get insights into how the industry uses alternate platforms to market its products.

## Methods

### Digital platform selection

**Fig. 1:**
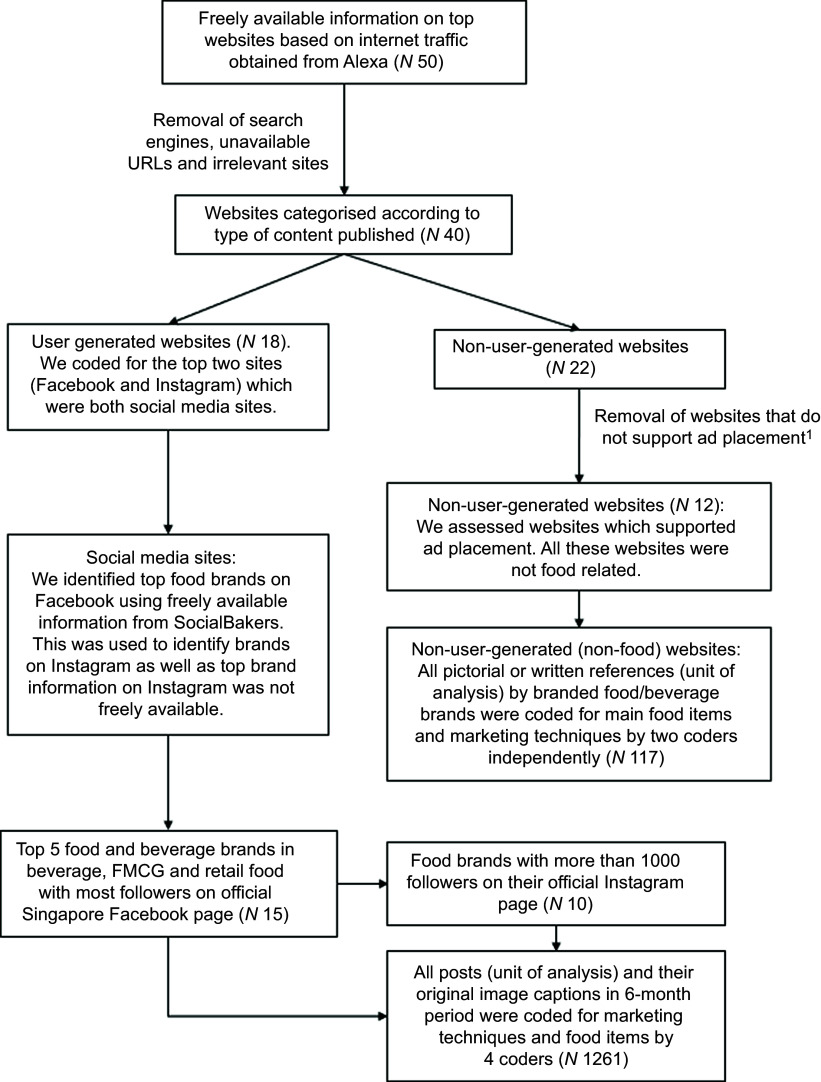
Flow diagram for the selection of digital media platforms and advertisements. ^1^Websites removed included banking websites, subscription websites, etc.

We extracted freely available information from Alexa^(21)^ in April 2018 on the top fifty websites in Singapore according to internet traffic rank, which was one of the few sources of freely available information in 2018. We excluded pornography sites, search engines (e.g. Google), sites with no advertising content (e.g. Wikipedia) and sites with an unavailable URL, after which forty sites remained. Although search engines ranked top of the list, ad exposure is largely determined by the search terms entered by individual users^(22)^, so it was not considered possible to develop a suitable coding framework. We then classified sites according to whether or not the websites allowed users to post content. Twenty-two of the forty websites did not allow for posting primary content by its viewers (we termed these as non-user-generated content websites) and were all included in our sample (Fig. 1). Of the eighteen websites that presented content, which were created by users (user-generated content websites), we limited this to Facebook and Instagram, which were among the leading social media platforms in Singapore^(23)^. Other top user-generated content websites including YouTube, Reddit and Twitter were excluded due to study resource constraints.

### Advertisement sampling from websites

We used a standardised protocol adapted from other studies^(20,24)^ to capture advertisements presented by the websites. Specifically, for non-user content websites such as Yahoo, advertisements on pages within twenty mouse clicks from the homepage were included. If the website presented a food section, coders also navigated to the food section and retrieved food advertisements on pages found within twenty mouse clicks from the food section. Both embedded advertisements, that is, presented as images integral to the article, and banner advertisements were included. Every pictorial or written reference to a food or beverage brand on the website was also recorded. Videos were not observed on these sites. The twenty mouse clicks did not include clicking on embedded links of pictorial or written references to external webpages of food or beverage brands, which were also included. To avoid variations in ad presentation due to targeting, advertisement capture was done using incognito mode, after clearing the browser history. For Facebook, freely available information from the social media monitoring site SocialBakers^(25)^ was used to identify the top five brands with the most followers on their Singapore channel for each of the following three categories: retail food; beverages; and fast-moving consumer goods (FMCG) food^(25)^. As freely available information to identify top brands on Instagram was unavailable, we used the same brands for Instagram, focusing on those that had over 1000 followers on their official Singapore Instagram pages (*n* 10) (Fig. 1). The legitimacy of the companies’ Singapore Facebook and Instagram pages was ascertained by the presence of a ‘verified’ badge. All posts, including textual, pictorial and videos, and original image captions presented by the companies on their Facebook and Instagram pages over a 6-month period from 1 January 2018 to 30 June 2018 were included in the sample. Screenshots of the posts were captured to allow for checking of ad coding if required.

### Coding frameworks

#### Marketing techniques

For non-user-generated websites, coding methodology and marketing techniques for the marketing coding framework for non-food websites were adapted from a previous study conducted in New Zealand^(26)^. Two coders independently coded sites, one at a time, and discussed discrepancies and gaps before updating the framework iteratively and moving on to another site. Each post was coded for twelve marketing techniques including health claims, corporate social responsibility and price promotions (Table 1).

**Table 1:** Brand presentation and marketing techniques employed by posts of top twelve non-food and beverage websites in Singapore

Brand presentation and marketing techniques	*n*	%
Brand presentation		
Only pictorial reference to brand	25	21 %
Only written reference to brand	6	5 %
Both pictorial and written references to brand	79	68 %
Part of editorial content	87	74 %
Marketing techniques		
Photos	95	81 %
Links to brand’s websites	86	74 %
Health-related claims	27	23 %
New brand development	23	20 %
Events and/or festivals	22	19 %
Special price promotions, contests and/or giveaways	18	15 %
Videos	17	15 %
Cartoon characters, celebrities, sportspersons	11	9 %
Advercation^*^	10	9 %
CSR^†^ and/or philanthropy	5	4 %
Healthier Choice Symbol^‡^	3	3 %
Activities, games and/or quizzes	0	0 %

* Advercation is a marketing technique that could educate users about nutritional information, historical facts, sports information or details of products, while advertising.

† Corporate social responsibility and/or philanthropy.

‡ Healthier Choice Symbol is a front-of-pack endorsement from the Singapore Health Promotion Board, a statutory board tasked with managing national health promotion efforts.

For Facebook and Instagram, we based our marketing coding framework on those of other studies of these platforms^(18,20)^. The coding frameworks used for these platforms were more detailed reflecting the complexity of the material presented. Specifically, each post and caption were coded for the presence of twelve broad marketing categories (such as characters involved, sponsorships and/or partnerships, health claims description and brand benefit claims) and sixty-nine elements. Descriptive definitions with examples for each marketing technique are presented in online supplementary material, Supplemental Table 1. Four coders were trained to independently code for all food and beverage posts on Facebook and Instagram. To limit brand-specific inter-rater variability, each 6-month period was divided across four coders for each brand. Any uncertainties from coding were reviewed and addressed before coders moved on to another food brand, and the training guide was iteratively updated. For videos, coders stopped at each video frame to code for both marketing techniques and food items while watching the video, after which the video was reviewed to check for coding accuracy. The original image caption of the video was also coded for marketing techniques.

Screenshots of all images, original image captions and full videos were uploaded onto a Microsoft Access coding form. The number of followers of food and beverage companies was obtained on their Facebook and Instagram pages. The number of likes (where users show a positive reaction), number of comments (where users respond to a post), number of views (where users watch the video) and number of shares (where users broadcast the content to their own social networks) were tied to the individual post on Facebook and Instagram. The level of engagement with the advertisements on social media sites was determined by the number of likes, number of comments, number of shares and number of views. Information on the number of likes, comments, shares and views was recorded for all posts during the study period.

#### Food quality

Food items present in posts were coded for food type according to an adapted version of the WHO nutrient profile frameworks^(27,28)^. While we primarily used the nutrient profile framework for the Western Pacific region, for foods that were not specified, we referred to the Southeast Asian nutrient profile framework. Examples included the classification of fermented dairy product-based milk drinks under milk drinks and sweet desserts under chocolate and sugar confectionery. Further adaptations included more detailed categorisation of beverages into sugary beverages, sugar-free sweetened beverages, water/non-sweetened beverages and alcoholic beverages according to the sugar content and type of sweeteners present in the beverages. Composite dishes were grouped into Western-style prepared food and Asian-style prepared food. A category for health supplements, herbs and tonics was also included in the food nutrient profile framework. Descriptive definitions with examples for each food category are presented in online supplementary material, Supplemental Table 2. Foods were then broadly categorised as core foods, non-core foods or mixed dishes using a classification system adapted from the INFORMAS protocol^(29)^ based upon Singapore dietary guidelines^(30)^. Core foods are nutrient-rich foods that are recognised as foundational by national dietary guidelines such as eggs, fresh meat, fresh fruits, vegetables and cereals. Non-core foods are discretionary food items such as sweetened beverages and sweet and savoury snacks. Mixed dishes are referred to as composite dishes under the WHO nutrient profile framework^(27,28)^. We further classified the mixed dishes according to how they were prepared as Asian-styled prepared food (e.g. rice dishes), Western-style prepared food (e.g. burgers) and instant and convenience-packed food (e.g. instant noodles). For advertising messages on non-user-generated websites, only the main foods explicitly associated with food brands were included for coding. For Instagram and Facebook, similar to non-user-generated websites, we coded for foods whose brand identity was clear. However, in the context of social media sites of food companies, this covered all foods referenced in the post.

### Statistical analyses

Data on brand characteristics, engagement levels, marketing techniques and food classifications were summarised using averages and proportions as suitable. Analyses were performed using SPSS version 25.

## Results

### Non-user-generated websites

A large proportion of the popular non-user-generated websites were news or entertainment based. We observed 117 advertisements marketing foods or beverages on the twelve websites sampled, with an average of 10 per site (median 11 per site) (Table 2). The majority of the advertisements (74 %) on these sites were presented in the context of editorial content related to current events such as the move to phase out plastic straws at food outlets in Singapore (see online supplementary material, Supplemental Fig. 1) rather than a sponsored advert (Table 1). Links to the brand’s websites were present in 74 % of advertisements. Health claims such as ‘healthier option’ and ‘without any added sugar’ were used in around a quarter of advertisements; however, only a few had the government endorsed the Healthier Choice Symbol logo. New product launches and associations with contemporary events such as the 2018 FIFA World Cup or the 2018 North Korea–United States Singapore Summit were used in around one-fifth of advertisements. Mixed dishes which included Western-style prepared food such as burgers, fried chicken and fast-food meals and other non-core foods primarily marketing sweet snacks and beverages were present in the large majority (84 %) of advertisements (Table 2). Core food advertisements were in the minority (16 %), and these were fairly evenly distributed among water, unsweetened drinks, milk, breads, fresh meats, grains, fruit, vegetables and legumes (data not shown). About one in ten (*n*=10, 9 %) food and beverage advertisements were for alcoholic beverages.

**Table 2:** Characteristics of advertisements on top twelve non-food and beverages websites and on social media pages of fifteen major food and beverage companies in Singapore^*^

Name	Business type	Ads sighted per website, *n*	Number of food/beverage items coded	Food types^†^,^||^					
				Core food/ beverage		Non-core food/beverage		Mixed dishes	
				*n*	%	*n*	%	*n*	%
Non-food websites		*n* ^§^ 117	*n* 117	19	16 %	63	54 %	35	30 %
Yahoo	News/entertainment provider	19	19	4	21 %	5	26 %	10	53 %
Baidu	News/entertainment provider	0	0	0	0 %	0	0 %	0	0 %
Straits Times	News/entertainment provider	15	15	2	13 %	9	60 %	4	27 %
Channel News Asia	News/entertainment provider	19	19	3	16 %	11	58 %	5	26 %
HardwareZone	IT-oriented internet portal	0	0	0	0 %	0	0 %	0	0 %
NTD	Television broadcaster	3	3	1	33 %	1	33 %	1	33 %
The Epoch Times	News/entertainment provider	10	10	1	10 %	7	70 %	2	20 %
MSN	News/entertainment provider	20	20	6	30 %	10	50 %	4	20 %
Providr	Online magazine	19	19	1	5 %	10	53 %	8	42 %
QQ	Messaging service	0	0	0	0 %	0	0 %	0	0 %
Epoch Times	News/entertainment provider	12	12	1	8 %	10	83 %	1	8 %
Kiss Anime	Anime website	0	0	0	0 %	0	0 %	0	0 %
Average per site^¶^		10	10	2		5		3	
Median per site		11	11	1		6		2	
Social media sites^‡^		*n* ^§^ 1261	*n* 1699	229	13 %	984	58 %	486	29 %
MILO	Beverage food company	28	38	10	26 %	25	66 %	3	8 %
Coca-Cola	Beverage food company	61	56	4	7 %	48	86 %	4	7 %
NESCAFÉ	Beverage food company	6	6	0	0 %	5	83 %	1	17 %
100PLUS	Beverage food company	74	91	9	10 %	74	81 %	8	9 %
LiHO	Beverage food company	176	229	57	25 %	165	72 %	7	3 %
Sticky	FMCG** food company	61	57	0	0 %	57	100 %	0	0 %
Ben & Jerry’s	FMCG** food company	63	76	3	4 %	73	96 %	0	0 %
Ferrero Rocher	FMCG** food company	26	22	0	0 %	22	100 %	0	0 %
KitKat	FMCG** food company	22	21	2	10 %	19	90 %	0	0 %
Kinder Bueno	FMCG** food company	24	18	0	0 %	18	100 %	0	0 %
McDonald’s	Retail food company	131	235	30	13 %	113	48 %	92	39 %
Starbucks	Retail food company	133	141	13	9 %	114	81 %	14	10 %
foodpanda	Retail food company (food delivery website)	236	387	71	18 %	124	32 %	192	50 %
KFC	Retail food company	123	201	19	9 %	72	36 %	110	55 %
Subway	Retail food company	97	121	11	9 %	55	45 %	55	45 %
Average per brand^††^		84	113	1		66		32	
Median per brand		63	76	9		57		4	

* Non-food websites are presented according to the internet traffic rank; food and beverage companies are presented according to the number of followers on Facebook.

† Table percentage reflects row percentage.

‡ Facebook and Instagram posts were combined for social media sites.

§ Refers to total numbers.

|| Row percentages that add up to more than 100 % are where multiple food items were coded in a single post.

¶ Average per site was calculated by averaging values across twelve non-food websites.

** Fast-moving consumer goods refer to low-cost consumer goods, which tend to be sold at high volumes^(33)^.

†† Average per brand was calculated by averaging values across fifteen social media pages of food and beverage companies.

### Social media platforms of food and beverage companies

We observed 1261 Facebook and Instagram posts from the fifteen top food and beverages brands in Singapore across the twenty-five Facebook and Instagram sites, with an average of 50 posts per brand per site (0·3 posts per day) and a median of 36 posts per brand per site over the 6-month period (Table 3). About a third (*n* 372, 30 %) of the posts observed were videos. Retail food brands posted an average of 80 times and a median of 89 posts over the 6-month period (almost every other day) and were more active than beverage brands (average 38 posts; median 36 posts) and FMCG brands (average 28 posts; median 27 posts). Retail food companies had more posts on Facebook (average 112 posts; median 98 posts) as compared to Instagram (average 40 posts; median 36 posts) over the 6-month period, but this was largely comparable for beverage (average: 39 (Facebook), 38 (Instagram); median: 37 (Facebook), 30 (Instagram)) and FMCG companies (average: 26 (Facebook), 33 (Instagram); median: 26 (Facebook), 33 (Instagram)) (Table 3).

**Table 3:** Characteristics of 1261 Facebook and Instagram posts of fifteen major food and beverage companies in Singapore

Company*	Site	Number of posts, *n*	Total likes of posts, *n*	Average likes per post	Total comments on posts, *n*	Average comments per post	Total shares of posts, *n* ^†^	Average shares per post	Number of videos, *n*	Total views of videos, *n*	Average views per video
Beverages subtotal		345	84 919	246	7093	21	7536	39	96	3 674 726	38 278
100PLUS	FB	38	4545	120	291	8	167	4	24	462 781	19 283
	IG	36	2158	60	32	1	–	–	9	3114	346
Coca-Cola	FB	37	18 172	491	1094	30	1416	38	20	1 934 111	96 706
	IG	24	2449	102	197	8	–	–	13	10 189	784
LiHO	FB	87	12 529	144	3380	39	3336	38	5	102 730	20 546
	IG	89	32 168	361	653	7	–	–	9	14 819	1647
MILO	FB	27	8879	329	779	29	2309	86	12	964 888	80 407
	IG	1	134	134	5	5	–	–	1	1390	1390
NESCAFÉ	FB	6	3885	648	662	110	308	51	3	180 704	60 235
FMCG^‡^ subtotal		196	13 394	68	826	4	1600	12	55	507 970	9236
Ben & Jerry’s	FB	27	2063	76	334	12	915	34	14	201 686	14 406
	IG	36	4614	128	218	6	–	–	14	15 137	1081
Ferrero Rocher	FB	26	2039	78	35	1	500	19	10	79 649	7965
Kinder Bueno	FB	24	2119	88	110	5	97	4	1	4979	4979
KitKat	FB	22	591	27	73	3	66	3	16	206 519	12 907
Sticky	FB	31	483	16	27	1	22	1	0	–	–
	IG	30	1485	50	29	1	–	–	0	–	–
Retail food subtotal		720	488 980	679	101 098	140	114 826	205	221	13 973 412	63 228
foodpanda	FB	171	23 684	139	2766	16	940	5	31	162 648	5247
	IG	65	4394	68	163	3	–	–	6	2628	438
KFC	FB	98	86 030	878	15 632	160	34 273	350	49	4 276 710	87 280
	IG	25	17 491	700	353	14	–	–	4	21 306	5327
McDonald’s	FB	104	68 125	655	35 487	341	37 161	357	62	6 022 503	97 137
	IG	27	23 791	881	1091	40	–	–	18	176 215	9790
Starbucks	FB	89	114 441	1286	39 774	447	39 235	441	19	1 402 750	73 829
	IG	44	111 097	2525	2055	47	–	–	23	390 485	16 978
Subway	FB	97	39 927	412	3777	39	3217	33	9	1 518 167	168 685
Total		1261	587 293	466	109 017	86	123 962	140	372	18 156 108	48 807

FB denotes Facebook, and IG denotes Instagram.

* Instagram followers were under 1000 for some brands, and these were not included in our sample.

† Information of shares was not available for Instagram posts.

‡ Fast-moving consumer goods refer to low-cost consumer goods, which tend to be sold at high volumes^(33)^.

When we examined the levels of engagement (likes, comments, shares), we observed that the primary form of engagement was likes (average 416 likes per post; median 101 likes per post), followed by shares (average 140 shares per post; median 6 shares per post) and comments (average 55 comments per post; median 7 comments per post). Retail food brands also had more likes per post (average 679 likes per post; median 141 likes per post) than beverage brands (average 246 likes per post; median 78 likes per post) and FMCG brands (average 68 likes per post; median 36 likes per post). Average likes per post and median likes per post were higher on the Instagram platform for retail food brands (average: 974 (Instagram), 594 (Facebook); median: 514 (Instagram), 132 (Facebook)) and FMCG brands (average: 92 (Instagram), 56 (Facebook); median: 74 (Instagram), 14 (Facebook)). Median likes per post were higher on the Instagram platform for beverage brands (Instagram: 189, Facebook: 44), but average likes per post were comparable across Instagram and Facebook (Instagram: 246, Facebook: 246). Out of the fifteen top food and beverage brands in Singapore, Starbucks, a retail food brand, was found to have the highest number of average likes per post (Facebook: 1286, Instagram: 2525) and highest median likes per post (Facebook: 476, Instagram: 2218) on both Facebook and Instagram.

### Marketing techniques

Apart from branding elements that were present in all posts, the most prominent marketing technique was brand benefit claims (1075 posts, 85 %), which emphasised positive attributes of the product apart from health (Table 4). These attributes included highlighting sensory characteristics such as taste and aroma (see online supplementary material, Supplemental Fig. 2) or suggested uses such as for sharing and emotive claims invoking emotions such as happiness and excitement. Another popular marketing technique employed by companies was providing sources for more brand-related information via links to their websites or social media handles (926 posts, 73 %). Premium offers were more often used by retail food brands (63 %) and beverage brands (47 %) as compared to FMCG brands (28 %). The nature of the offers typically included limited-time offers, free delivery with a minimum spend and limited edition merchandise. Beverage brands used marketing techniques such as characters (40 %) and health claim descriptions (32 %) in a larger proportion of their posts as compared to FMCG (3 and 1 %, respectively) and retail food brands (3 and 5 %, respectively) (Table 4). Characters typically presented in posts by beverage brands included local celebrities from Singapore’s national public broadcaster (Mediacorp) and professional sportspersons such as Olympic gold medallist Joseph Schooling. Health-related claims in beverage brand posts emphasised benefits such as ‘healthier option’, ‘no added sugar’ and the use of sugar substitutes such as stevia. The Healthier Choice Symbol, a government-endorsed front-of-pack label, was observed in 20 % of beverage posts. Viral marketing elements were present in the majority of the posts (81 %) by beverage brands but less frequently used by FMCG (63 %) and retail food brands (30 %). Sponsorship and/or partnerships were more prominent for beverage (30 %) (see online supplementary material, Supplemental Fig. 3) and retail food brands (28 %) than FMCG brands (3 %). Major sponsorships and partnerships included sponsorships by 100PLUS with Singapore celebrities, and Coca-Cola as a sponsor of the 2018 FIFA World Cup. Across all food and beverage brands, user-generated content (UGC) and corporate social responsibility or philanthropic marketing techniques were rarely employed, with one exception. About 40 % of Ben & Jerry’s posts featured corporate social responsibility or philanthropic marketing elements emphasising the fair trade and sustainability of ingredients used and social premiums given to their small-scale farmers worldwide (see online supplementary material, Supplemental Fig. 4). Online supplementary material, Supplemental Table 3 contains the details of marketing elements observed on the 1261 advertisements observed on Facebook and Instagram. Although we were not able to identify if food advertisements were specifically targeted at children, marketing techniques such as cartoon characters were observed in a few posts (five posts, 0·3 %).

**Table 4:** Marketing techniques observed on Facebook and Instagram posts of fifteen major food and beverage companies in Singapore^*^^,^^†^^,^^‡^

Brands	Characters^§^		Events and/or festivals		Premium offers		Health-related claims		Brand benefit claims		CSR^||^		Engage-ment		Viral marketing		Sponsorship and/or partnership		Links^¶^		Advercation		UGC**	
	*n*	%	*n*	%	*n*	%	*n*	%	*n*	%	*n*	%	*n*	%	*n*	%	*n*	%	*n*	%	*n*	%	*n*	%
Beverage subtotal																								
(*n=* 345)	138	40	171	50	161	47	109	32	272	79	2	1	149	43	280	81	102	30	233	68	17	5	12	3
100PLUS	39	53	29	39	26	35	50	68	52	70	0	0	24	32	58	78	25	34	45	61	4	5	5	7
Coca-Cola	36	59	46	75	42	69	20	33	46	75	0	0	34	56	48	79	24	39	49	80	1	2	0	0
LiHO	45	26	86	49	70	40	14	8	145	82	2	1	66	38	164	93	33	19	111	63	11	6	6	3
MILO	17	61	10	36	20	71	24	86	24	86	0	0	22	79	8	29	20	71	22	79	1	4	1	4
NESCAFÉ	1	17	0	0	3	50	1	17	5	83	0	0	3	50	2	33	0	0	6	100	0	0	0	0
FMCG^††^ subtotal																								
(*n* =196)	5	3	80	41	54	28	2	1	167	85	25	13	72	37	124	63	5	3	147	75	8	4	11	6
Ben & Jerry’s	0	0	23	37	29	46	2	3	60	95	25	40	41	65	37	59	3	5	43	68	3	5	3	5
Ferrero Rocher	0	0	20	77	0	0	0	0	9	35	0	0	9	35	4	15	0	0	26	100	0	0	0	0
Kinder Bueno	0	0	2	8	1	4	0	0	20	83	0	0	12	50	22	92	0	0	24	100	4	17	0	0
KitKat	1	5	8	36	17	77	0	0	21	95	0	0	3	14	2	9	2	9	22	100	1	5	0	0
Sticky	4	7	27	44	7	11	0	0	57	93	0	0	7	11	59	97	0	0	32	52	0	0	8	13
Retail food subtotal																								
(*n=* 720)	25	3	163	23	456	63	39	5	636	88	10	1	405	56	217	30	202	28	546	76	55	8	2	0
foodpanda	3	1	42	18	143	61	9	4	219	93	4	2	165	70	6	3	142	60	199	84	30	13	0	0
KFC	12	10	35	28	91	74	5	4	116	94	3	2	74	60	106	86	10	8	82	67	9	7	2	2
McDonald’s	7	5	61	47	93	71	8	6	120	92	2	2	68	52	28	21	27	21	94	72	8	6	0	0
Starbucks	3	2	15	11	97	73	2	2	110	83	1	1	39	29	59	44	14	11	79	59	3	2	0	0
Subway	0	0	10	10	32	33	15	15	71	73	0	0	59	61	18	19	9	9	92	95	5	5	0	0
Total	168	13	414	33	671	53	150	12	1075	85	37	3	626	50	621	49	309	25	926	73	80	6	25	2

* Row percentages that add up to more than 100 % are where multiple marketing techniques were coded in a single post.

† Branding as a marketing technique was observed in all 1261 posts.

‡ All data in table are presented as *n* (%).

§ Characters involved.

|| Corporate social responsibility and/or philanthropy.

¶ Links and/or multimedia.

** User generated content.

†† Fast-moving consumer goods refer to low-cost consumer goods, which tend to be sold at high volumes^(33)^.

### Types of foods advertised

Overall, the predominant foods advertised were non-core foods or beverages (58 %), followed by mixed dishes (29 %) and core foods or beverages (13 %) (Table 2). This varied by company type and the details of food categories by companies are presented in online supplementary material, Supplemental Table 4. The marketing portfolio of beverage and FMCG companies comprised mainly of non-core foods, which ranged from 66 to 100 % of foods advertised. Retail food brands (McDonald’s, foodpanda, KFC and Subway) mostly displayed similar proportions of non-core foods and mixed dishes.

More than half of the core food displayed were fruits, vegetables and legumes (data not shown) and found at foodpanda (31 %), LiHO (25 %) and McDonald’s (13 %) sites. Posts showing core food were absent for nearly a quarter of the food and beverage brands (NESCAFÉ, Ferrero Rocher, Kinder Bueno and Sticky). A low percentage of food advertisements from the top fifteen food and beverage brands displayed core food, except for LiHO (26 %) and MILO (25 %). Similar to non-user-generated websites, most of the non-core food displayed on social media platforms were sweetened beverages (38 %) and sweet snacks (38 %) (data not shown). Mixed dishes comprising primarily Western-style fast food (burgers, fast-food meals and fried chicken) and Asian-style prepared food (sushi, noodle dishes and rice dishes) were mostly observed in retail food brands posts such as foodpanda (40 %), KFC (23 %) and McDonald’s (19 %). The majority of mixed dishes (75 %) present in the food and beverage brands were Western-style dishes (data not shown) and no mixed dishes were observed in posts by FMCG brands. Alcoholic beverages were observed in a few of the Facebook and Instagram post (*n* 4).

## Discussion

We aimed to systematically describe the digital food and beverage marketing landscape in Singapore. For this purpose, we evaluated the number and nature of food advertisements on popular non-food websites and on social media sites of food and beverage brands. We observed 117 advertisements on 12 non-food websites and 1261 advertisements on the 25 social media sites of food and beverage companies. A vast majority of the food advertisements on both non-food websites and social media sites were for non-core foods or mixed dishes, with links for further information commonly used as a marketing strategy. For non-food websites, advertisements were often presented as a part of editorial content. For social media sites, variations in marketing techniques by food category were observed. Retail food brands often used premium offers while beverage brands commonly employed viral marketing and more frequently made health-related claims. A small proportion of the advertisements (7 %) had the government endorsement of the Healthier Choice Symbol, but other brand benefit claims focusing on the sensory aspects of the foods were widely employed.

Similar to a study on food marketing to New Zealand children and adolescents^(26)^, we found a limited suite of marketing techniques used on non-food websites. Most of the food marketing we found on these sites was included as editorial content on news/entertainment provider websites, and most articles contained links to food/beverage brand websites. It is uncertain why this type of content exists and whether there is any influence of or partnership with the food industry in generating this content. However, as over half of the promoted foods were non-core, ‘unhealthy’ foods, this area requires more attention from the public health community. As the study does not discern behaviourally targeted advertising, sponsored food and beverage advertisements targeted at young people specifically would not have been identified.

As observed in other studies^(33)^, we found that non-core food was sighted in the majority of the food advertisements on social media platforms Facebook and Instagram. While food companies may pledge, or be working towards, improving the nutritional quality of their product portfolio^(31)^, the majority of the marketed foods were still non-core foods and of poor nutritional value.

The average volume of social media posts on a brand’s page was 50 over the 6-month period, suggesting that followers of these pages would be highly exposed to marketing messages. This equates to about a post every other day, the same amount reported in a New Zealand study of forty-five brands Facebook pages^(19)^.

The dominant use of brand benefit claims highlighting the sensory appeal and convenience of foods as a marketing strategy is well aligned with key personal drivers of food intake^(34)^. In contrast, only beverage brands used health-related claims as a marketing strategy frequently. Singapore’s Healthier Choice Symbol programme allows beverages containing less than 6–7 g of sugar per 100 g to display the symbol and make the claim ‘Lower in Sugar’^(35)^. Out of the 345 posts by beverage brands, a similar proportion of Facebook (30 %) and Instagram posts (23 %) alluded to the relative healthfulness of their beverages by referencing the use of sugar substitutes, using terms such as ‘reduced’ or ‘no sugar’ or displaying the Healthier Choice Symbol (5 %). This finding on the use of health-related claims by beverage brands is in contrast to a content analysis of sugar-sweetened beverage brand pages on Facebook in Australia, in which fewer than 5 % of posts referred to ‘reduced’ and/or ‘no sugar’^(36)^. The types of beverage also differed, in that in the Australian study the top brands were soft drinks and energy drinks, whereas in our study in addition to these, the top brands included a malted beverage brand (MILO) for which 86 % of posts featured a health-related claim (see online supplementary material, Supplemental Fig. 5). Nonetheless both studies included Coca-Cola. In our study, a third of Coca-Cola posts included health-related claims, which were related to the sugar content, whereas only 3 % of Coca-Cola posts in the Australian study featured low-sugar products. The emphasis on marketing beverages as healthier may be a response to Singapore government actions and health promotion efforts that have focused on lowering the consumption of sugary drinks. This suggests that the marketing techniques of an individual brand can vary greatly by country, making global monitoring more challenging.

One cross-country study compared marketing techniques and contents in high (the USA and Germany), upper-middle (China and Mexico) and lower-middle-income countries (the Philippines and India)^(37)^. In this study, brands (Coca-Cola, McDonald’s, KFC) featured diet/healthier products more frequently in high-income countries as compared with in lower-middle-income countries, while they featured philanthropy more frequently in lower-middle-income countries. It is unsurprising that brands’ marketing techniques differ by country, with local consumer preferences and social norms, different promotion concepts are needed in different settings^(38)^. Localisation refers to this tailoring of content for an individual country, and it is suggested that the amount of effort that goes into localisation is governed by the size of the market^(38)^. While it makes monitoring challenging, cross-country comparisons can provide insights to policymakers and public health professionals about the impact of advertising restrictions.

Our observation that retail food companies are more active in social media spaces, with posts and videos that have higher reach, and engagement, may be related to the dietary behaviours of our population and the marketing strategies employed by these companies. Eating out is a social norm in Singapore, and, in 2010, over 75 % of adults reported usually eating out for at least one of three meals^(39)^. Although people typically eat out at local hawker centres/canteens, eating at fast-food restaurants is more common in younger adults (18–21 years) with about 70 % eating at these venues on a weekly basis^(39)^. In 2020, total sales of this sector were about 1·4 billion SGD^(40)^. The use of premium offers widely employed by retail food companies may therefore be a way of reducing the cost differential between fast foods and the less expensive hawker foods. Singaporean young adults reported actively searching for and being more engaged with food social media posts that highlighted price promotions, which were shared with their networks, and this often resulted in dining out at these eateries^(41)^. Although young adults in Singapore are aware of the commercial intent of advertising, the social pressure of conforming to micro-trends created by the food industry using strategies like limited-timed or premium offers was viewed as being persuasive^(41)^. As young adults who are followers of food brands on social media are more likely to report food cravings after seeing food ads^(42)^, it would be of interest to see if they are key nodes in their social network that amplify the reach of food companies on social media. While we did not specifically evaluate variation in engagement levels by marketing strategies, it would be of value for future studies to examine this and also assess whether this varies by type of food.

In Singapore, food advertisements directed primarily to children of 12 years or younger must meet specific nutrition criteria to be broadcast^(43)^. For online media, this was limited to a few Singapore domain websites directed primarily to children^(43)^. However, more recent efforts include mandatory nutrition labelling and banning the advertisement of sugary drinks, including digital spaces, which were implemented in December 2022^(44)^. A broader policy approach was approved in the UK in June 2021^(45)^, whereby companies cannot pay for advertising foods high in sodium, fat and sugar on online spaces. While these guidelines cover in-feed advertising on social media, company-owned media such as their own social media pages or websites are exempt. This exemption was to allow companies to share information about their products with people who wished to view this information by actively visiting the company pages. However, our results show that posts on companies’ social media pages are predominantly for non-core foods and are widely shared. This suggests that people may be inadvertently exposed to posts of unhealthy foods from their social networks, and some regulation around foods marketed on company-owned media should also be considered.

Instagram is often considered to appeal to a younger demographic and has been reported to facilitate a higher engagement rate between brands and consumers than Facebook^(20)^. This was also demonstrated in our study for the brands LiHO, McDonald’s, Starbucks and Sticky. Instagram is also considered an ideal platform for advertisers to encourage followers to create user-generated marketing content with the use of hashtag campaigns^(46)^, and these are more likely to be found in posts featuring adolescents as compared with posts featuring adults^(33)^. We found few instances of user-generated content, and it is unclear whether this is specific to our setting or the brands sampled. However, engagement techniques such as promotions/contests were prominent, and this marketing strategy has been observed in a number of other studies^(36)^.

This is one of the few studies to comprehensively characterise the marketing techniques employed by food and beverage companies and the nature of the foods marketed on digital platforms. We evaluated the marketing techniques of major food industry players across digital platforms, allowing comparisons by platforms. We used protocols established in the literature, facilitating the comparison of our results with those from other studies. Detailed analyses of marketing techniques using the coding framework provided deeper insights into the marketing techniques commonly employed by food and beverage companies on their social media posts, as well as the current digital food and beverage marketing landscape. This highlights the lack of action towards marketing regulations in the Western Pacific region, which serves to inform further policy discussion to control marketing as well as reinforce the importance of monitoring in the online digital space^(14)^.

Our study also had several limitations. The study did not identify the target audience of advertising as the content was publicly shared with followers of the food and beverage brands’ webpages, Facebook and Instagram. All users regardless of whether they were followers could access the same posts through searching online. With Facebook’s and Instagram’s algorithms, paid advertisements and sponsored posts are incorporated into users’ newsfeeds, whether or not they follow the brand. Although the brand’s official Singapore Facebook and Instagram pages were sampled in this study, there was a lack of freely available information on whether the brands were engaging with users based locally in Singapore. The number of followers on Singapore-based pages, while providing an estimation of social media outreach by brands, may not be limited to only Singapore residents. Although posts that exist for a longer time have more opportunity for engagement, an aspect that was not accounted for in this study, most brand engagement is likely to occur relatively soon after posting^(47)^. Other operational characteristics that may influence engagement including publication time, location and length of post^(48)^ are not considered and can be investigated in future studies. As this study utilises data from the pre-pandemic period, these numbers may not fully reflect the current activity levels of food companies on social media sites. Time spent by consumers in Singapore on social media platforms has doubled from 2 h pre-pandemic to 4 h post-pandemic^(23)^, and the online food delivery market in Singapore has increased more than 2·5 times from 589 million USD in 2019 to an estimated 1611 million USD in 2024^(49)^. While major food companies such as Coca-Cola, Nestle and Unilever have pledged to become more responsible in marketing unhealthy foods in the digital space^(50)^, this pledge is directed towards children under 13 years of age. Although not strictly enforced, children 13 years or younger are not legally allowed to have accounts on social media sites like Instagram and Facebook in Singapore, so it remains unclear how this pledge is applied or implemented on these popular platforms and further research is warranted.

Similar to other studies of this type, we used food nutrient profile frameworks to categorise foods based on their nutritional quality rather than calculating the nutritional composition of the foods marketed. The study was dependent on the social media monitoring site SocialBakers in identifying the top food and beverage brands in Singapore on social media sites such as Facebook and Instagram. The information on the top five beverage, FMCG and retail food brands was based solely on the total number of fans the brand has on Facebook in Singapore. Due to the limited social media monitoring sites available online, the reliability of top five beverage, FMCG and retail food brands in Singapore from SocialBakers could not be verified. As Facebook users are now able to receive updates on their newsfeed without liking the brand’s Facebook page, the number of users exposed to the brands’ marketing could be underestimated in this study.

### Conclusions

Monitoring of digital marketing activities on non-food websites and social media platforms showed that major companies in the food and beverage industry use multiple marketing techniques to promote largely non-core, ‘unhealthy’ foods on Singapore digital platforms. Commonly used marketing techniques included emphasising the product’s benefits, displaying links and/or social media handles and providing premium offers. As food companies move away from traditional static marketing, food advertising is more targeted and integrated into individuals’ newsfeeds on social media. In addition, food-related posts are widely shared. Increased incidental exposure to digital food advertising calls for policymakers to implement policies or guidelines to regulate food and beverage advertising on the internet. Given the lack of data on marketing techniques within digital environments in non-western countries, more research and similar environmental scans in these settings are urgently needed. Furthermore, continued monitoring of the online marketing of unhealthy foods and beverages in Singapore will be essential to evaluate the impact of new and future policies on restricting advertising in Singapore’s digital food landscape.

## Supporting information

Chua et al. supplementary material Chua et al. supplementary material

## Supplementary material

For supplementary material accompanying this paper, visit https://doi.org/10.1017/S1368980024002428.
