# Supplementary material for: Characterising the extent and nature of digital food and beverage marketing in Singapore: a descriptive study
Source: Public Health Nutr. 2024 Dec 12;28(1):e14. doi: 10.1017/S1368980024002428 (PMC11822605; doi:10.1017/S1368980024002428)
Supplement: Chua et al. supplementary material [file S1368980024002428sup001.docx]

**Multimedia Appendix 1**

Supplementary Figure 1. Example of editorial content from non-user generated website (Yahoo)


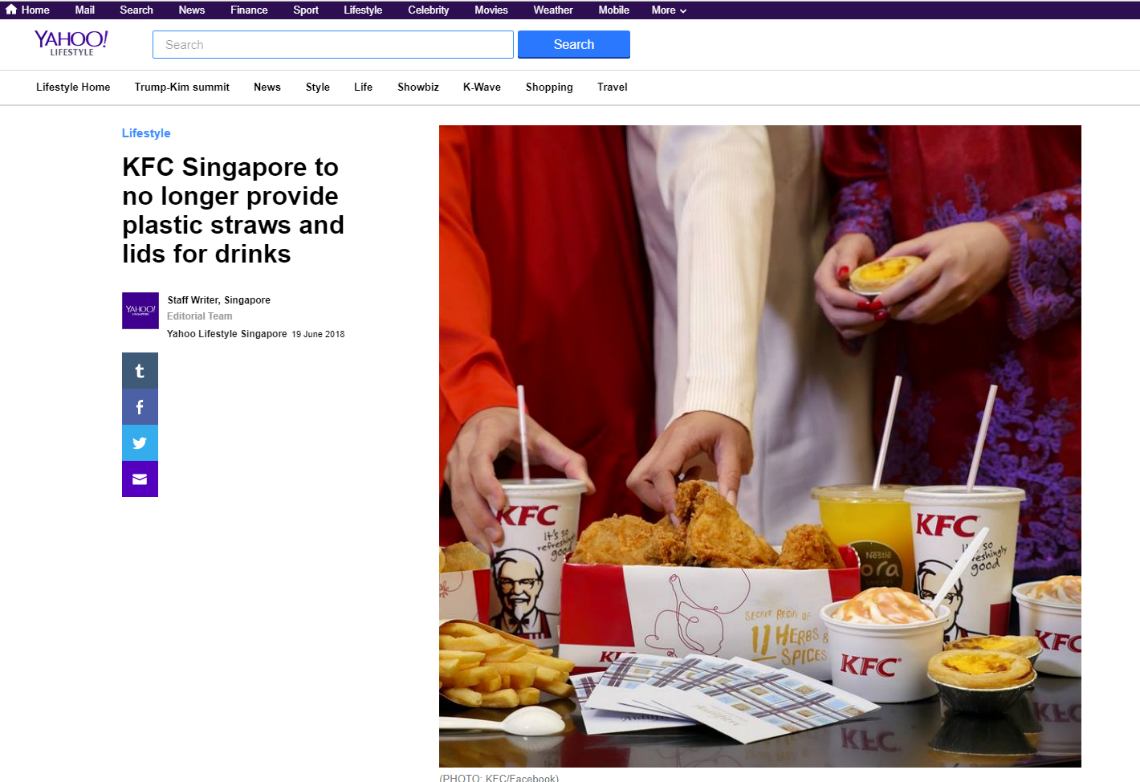


Supplementary Figure 2. Example of brand benefit claims from LiHO official Singapore Instagram page


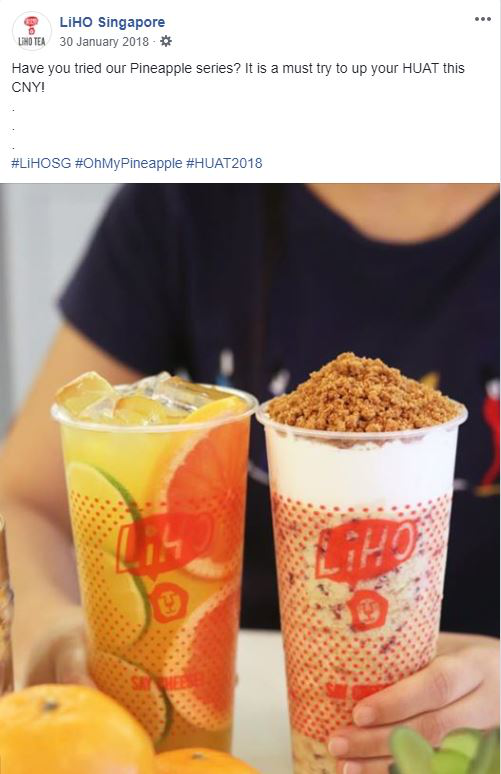


Supplementary Figure 3. Example of sponsorship and/or partnership from Coca-Cola official Singapore Facebook page


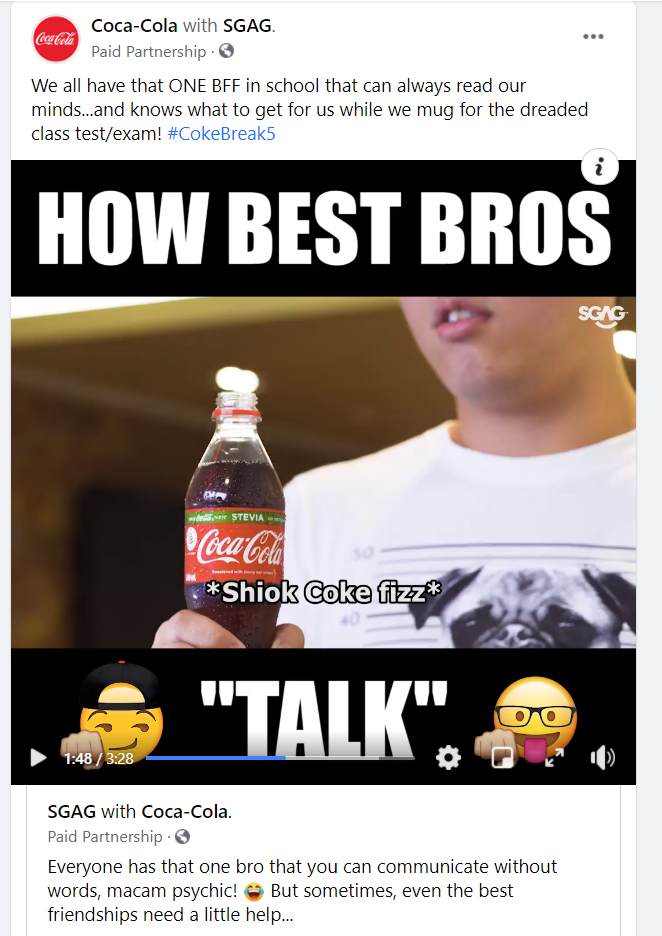


Supplementary Figure 4. Example of corporate social responsibility and/or philanthropy from Ben & Jerry’s official Singapore Facebook page


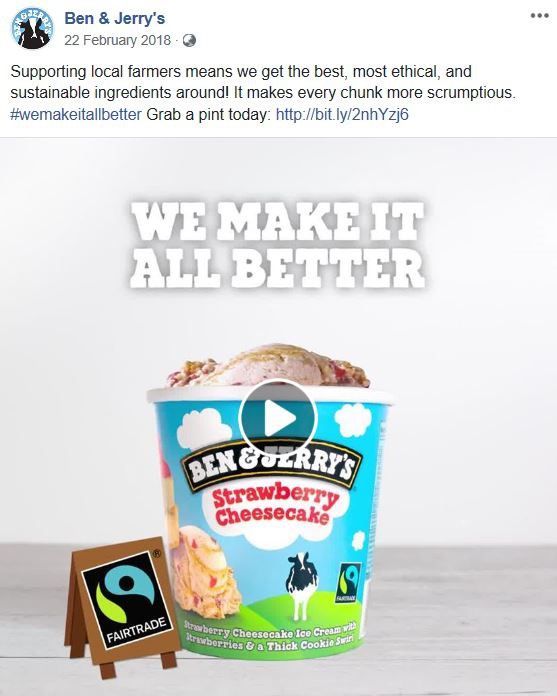


Supplementary Figure 5. Example of health claims from MILO official Singapore Facebook page


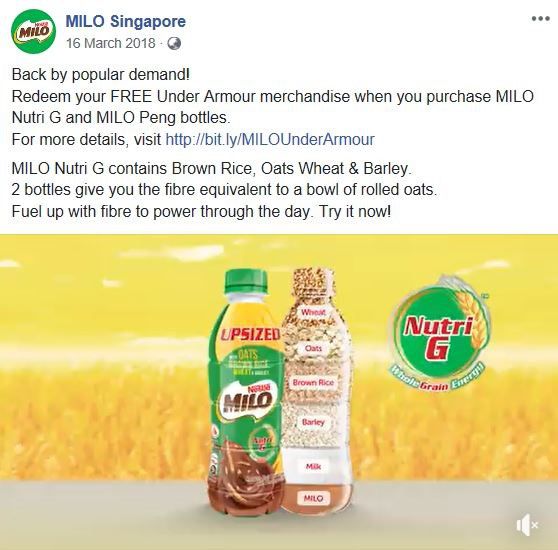


Supplementary Table 1 Definition of marketing techniques

| Category | Marketing techniques | Definitions |
| --- | --- | --- |
| Advercation (merged) | Advercation (details on product) | Type of advercation that educates users about the brand's products (i.e. origins of ingredients, production method, assembly process, etc.) while advertising |
| Advercation (merged) | Advercation (general nutrition) | Type of advercation that educates users on the nutritional information of the product while advertising |
| Advercation (merged) | Advercation (historical facts) | Type of advercation that educates users about practices of the past, past historical events, etc. while advertising |
| Advercation (merged) | Advercation (other) | Other types of advercation that do not fall under historical facts, general nutrition, sports information or details on products, while advertising |
| Advercation (merged) | Advercation (sports information) | Type of advercation that educates users on how a game is played, history of the game, athlete’s profile, etc. while advertising |
| Advercation (merged) | Inclusion in recipe | Advertising of products/ companies within a recipe |
| Brand benefit claims | Convenience | Verbal or written remarks of how a product brings about convenience, includes inferences made (i.e. a video showing the invention of smaller packages of a product can be inferred as convenience) |
| Brand benefit claims | Emotive claims | Claims that certain emotions and experiences will be evoked along with the use of the product or brand (i.e. fun, feelings, popularity) |
| Brand benefit claims | New brand development | Developed from research & development (mix & match of old products excluded) |
| Brand benefit claims | Puffery | Exaggerated or subjective claims of being better than/ advantageous over other brands in certain aspect |
| Brand benefit claims | Sensory based characteristics | Characteristics of a product based on its taste, texture, appearance and/or aroma mentioned or shown via visual effects |
| Brand benefit claims | Suggested use | A time of the day, mood, or environment, etc. that the food brand recommends using or consuming its product in |
| Brand benefit claims | Suggested users | Intended consumers of product are children or whole family |
| Branding | Brand name | A name given by the founder or which a company registers itself under |
| Branding | Branded characters | Any characters featured on the page developed by the brand |
| Branding | Colours | Colours that are visually associated or representative with/of the brand. (i.e. McDonald's brand colours are yellow and red) |
| Branding | Jingle | Any form of short tune developed by and is distinctive to the company |
| Branding | Logos | Any symbol or graphic that identifies the brand |
| Branding | Slogans | A short and striking phrase used by the brand as a brand identifier |
| Branding | Wordplay | Use of product name and/or brand to create a pun, with intended humour |

| Characters involved | Awards | A prize, certificate or other mark of recognition given in honour of an achievement |
| --- | --- | --- |

| Characters involved | Cartoon characters | Third-party cartoons and characters, including characters from films, books, television programs, and the Internet |
| --- | --- | --- |
| Characters involved | Celebrities | People with an entertainment or media profile, excluding athletes |
| Characters involved | Community | Ordinary people without an entertainment, sports, or media profile |
| Characters involved | Influencers | People with a following in a particular niche (active engagement with audience) or the power to affect purchase decisions of others because of his/ her authority, knowledge, position, or relationship with his/her audience |
| Characters involved | Kids | Children featured or involved in the promotional content of a product |
| Characters involved | Movie-tie in (characters) | Characters or scenes from movie releases included in the content of the advertisement |
| Characters involved | Other experts | People with credentials in their field of expertise. (i.e. health professionals, chefs, doctors) |
| Characters involved | Sportsperson | Any person (adult/ child) profiled for their athletic or sporting achievements |
| Characters involved | TV show-tie in (characters) | Characters or scenes from locally based TV shows included in the content of the advertisement |
| CSR^a^ and/or philanthropy | Corporate social responsibility | Ethical or sustainable initiatives (non-monetary based) |
| CSR^a^ and/or philanthropy | Philanthropy | Charitable work done |
| Engagement | Activities, games and/or quizzes | Posts that prompt or encourage participation & interaction among users (i.e. MCQ, open ended answers, quizzes, downloads & pop-ups) |
| Engagement | Advergaming | Advertising through game applications/ images (i.e. computer games) |
| Engagement | Community engagement and/or challenge | Any form of outreach to ordinary people without an entertainment, sports, or media profile (i.e. street interview) |
| Engagement | Storyline | A developmental narrative told using characters |
| Engagement | Conversations | Page administrator responds to page member's posts and/or comments |
| Engagement | Polls | Activities that involve taking sides |
| Events and/or festivals | Sports, community, historical and any festivals | Includes any sports, community, historical, etc. events that the brand is involved in. |
| Health claim description | General health claims | Broad health statements about a product (i.e. healthy diet, nutritious) |
| Health claim description | Health related ingredient claims | Presence of healthy ingredients (i.e. wholegrain) or lack of unhealthy ingredient (ie.no MSG, no artificial colouring). Exclude antioxidants. |
| Health claim description | Healthier choice symbol | Logo is present/ seen on packaging |
| Health claim description | Nutrient and other function claims | Information about the benefits of any particular nutrient found in the product (i.e. calcium is beneficial for bone health) |
| Health claim description | Nutrient comparative claims | Use of comparative words to describe the nutrient present in the product (i.e. reduced fats) |
| Health claim description | Nutrient content claims | Describing the nutrients present in the product (i.e. low fat, zero sugar, antioxidants) |
| Health claim description | Other (health) claims | Additional health claims not related to the nutrients and/or ingredients present in the product (i.e. organic/ no MSG) |
| Health claim description | Reduction of disease risk claims | Product claims to reduce the chances of developing a disease |
| Links and multimedia | GIFs or boomerang | Moving images |
| Links and multimedia | Links shared | Links to external pages or additional content |
| Links and multimedia | Photos | JPEG/PNG file format of the food products (includes those available for downloads) |
| Links and multimedia | Videos | Videos or slideshows of food products (includes those available for downloads) |
| Links and multimedia | Website communities | Post photos, videos, etc. made by people on brand’s webpages, forums, etc., excludes sponsored or commentaries done by publications |
| Premium offers | 2 for 1 deals | With every item purchased, the same item or an item of equivalent price will be given for free |
| Premium offers | Contests and/or giveaways | An entry that the community participates in to win a prize; giveaways include product samples or other items that are given upon winning the contest, after completing certain minimal requirements or with purchase, etc. |
| Premium offers | Exclusive offers for people who like/comment | Offers (i.e. price discounts) that are only given to those users who had completed minimal actions (i.e. liking or commenting on the page) as requested by the brand |
| Premium offers | Free extra ingredients | An increase in the quantity/ ingredient contents of products as compared to before (i.e. free 30% more coffee, more malt) |
| Premium offers | Game and app downloads | Any mention of apps that can be downloaded from the app store on computer or mobile devices, links to any smartphone apps and/or apps embedded in the Facebook page are included |
| Premium offers | Gifts or collectables | Items that are valued and sought by collectors |
| Premium offers | Limited edition merchandise/products | Limited edition' specifically mentioned as a verbal or written remark |
| Premium offers | Membership or loyalty rewards | Mention of premium offers specifically for members to enjoy exclusive offers, promotions and/or accumulate loyalty points |
| Premium offers | Price discounts, offers & rebates | Promo codes, limited time offers, vouchers, rebates, cashbacks, discounts, etc. |
| Premium offers | Seasonal products | Products created specifically for festivals or different seasons |
| Sponsorships and/or partnerships | Partnerships | An event that other brands or services are partnered with the brand of interest to offer mutual benefits for both parties, excludes charitable organization |
| Sponsorships and/or partnerships | Sponsorships | An event that other brands or services are clearly funded for by brand of interest to offer mutual benefits for both parties, excludes charitable organization. |
| Viral marketing | Follow | Cues to nudge users to follow the webpage |
| Viral marketing | Like | Cues to nudge users to like the webpage |
| Viral marketing | Share/post | Cues to nudge users to share/ post a post on the webpage |
| Viral marketing | Tag | Cues to nudge users to tag the webpage/ friends on the brand's webpage |
| Viral marketing | Hashtag | Cues to hashtag and/or # symbol representative of a hashtag found in posts, videos, etc. |

^a^ Corporate social responsibility

Supplementary Table 2 Food classification

| Food classification | Food category | | Examples of food included in category | Examples of food not included in category |
| --- | --- | --- | --- | --- |
| Non-core food | 1 | Chocolate and sugar confectionery, energy bars, and sweet toppings and desserts | Chocolate (including dark, white and milk chocolate), chocolate spread, snack bars (e.g. granola and muesli bars), hard/chewy candy, table sugars, spreads including peanut butter, marmalade, and jams, chewing gum, caramels, soft jellied candies, marshmallows, honey, puddings, cream desserts, sugar syrup, bubble tea toppings (black/brown/golden pearls, taro balls, aiyu jelly etc.) | Chocolate-flavoured breakfast cereals (Category 6), cakes and pastries, biscuits and other baked goods covered in chocolate, flour-based confectionery (Category 2) |
| Non-core food | 2 | Cakes, sweet biscuits and pastries, other sweet bakery products, dry mixes for making such food | Breakfast biscuits, cakes, cookies, pies, doughnuts, sweet rolls, muffins, macaroons, biscuits, pancake (ready–to-eat form) and other sweet flour-based confectionery | Bread products (Category 11) |
| Non-core food | 3 | Savoury snacks | Popcorn and maize corn, nuts, and mixed nuts (including with fruit content), seeds (i.e. sesame seeds), savoury biscuits, crackers, pretzels, other snacks made from rice, maize, wheat, dough, or potato (i.e. chips, crisps), pork and chicken rind, processed seaweed, fish-based snacks |  |
| Non-core food | 4 (a) | Juices | 100% fruit and vegetable juices prepared from direct extraction or fruit juice concentrates reconstituted with water only. Does not contain added sugar. Includes unsweetened fresh coconut juice. | Sugar cane juice, powdered juices, fruit juice concentrates reconstituted by adding sugar (Category 4d) |
| Core food | 4 (b) | Milk drinks | Milk, butter milk, fermented dairy-based milk, flavoured dairy based milk, (e.g. chocolate milk, strawberry milk, whey-based drinks), reconstituted powdered milk, almond, soya, rice and oat milks, goat milk, evaporated milk, condensed milk, sweetened creamer, evaporated milk, smoothies |  |
| Non-core food | 4 (c) | Tea and coffee | Caffeinated tea and coffee. Tea (including instant and premixed tea with caffeine), coffee (including instant and premixed coffee). Includes drinks made from tea leaves and coffee beans | Fruit tea without sugar (e.g. chamomile, strawberry tea, chrysanthemum tea), unsweetened herbal tea (Category 4f) |
| Non-core food | 4 (d) | Sugary beverages | Soft drinks/sodas, bubble tea, sugar cane, energy drinks, smoothie, drinks reconstituted from syrups, powdered juice, flavoured water, juice drinks, reconstituted chocolate or malted powdered drinks (e.g. Milo, Ovaltine), drinking yogurt, cultured drinks. Includes drinks made with honey and added table sugars/ syrups (e.g. fructose/sucrose), milkshake | 100% fruit and vegetable juices (Category 4a), milk drinks (Category 4b) |
| Non-core food | 4 (e) | \| [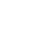](https://outlook.office.com/owa/?path=/attachmentlightbox)Sugar-free sweetened beverages \| \| --- \| | Beverages with artificial (e.g. aspartame, acesulfame potassium) and natural sweeteners (e.g. stevia) | Beverages with added sugar (Category 4d) |
| Core food | 4 (f) | Water/non-sweetened drinks | Non-caffeinated. Sparkling water, unsweetened herbal tea, fruit tea without added sugar, mineral water (including aerated) |  |
| Non-core food | 4 (g) | Alcoholic beverages | Drinks containing alcohol. Wine, beer, whiskey, vodka, spirits |  |
| Non-core food | 5 | Edible ices | Ice cream, iced lollies and sorbets, frozen fruit juices, frozen yogurt |  |
| Core food | 6 | Breakfast cereals | Chocolate breakfast cereals, oatmeal, mueslis, cornflakes, granola |  |
| Core food | 7 | Yogurts, cream, other similar foods | Yogurt, cheese-based and other yogurt substitutes, fermented milk, curds, whipped cream, cheese foam | Milks and unsweetened milks (Category 4b), drinking yogurt, cultured drinks (Category 4d) |
| Core food | 8 | Cheese | Unripened or ripened cheese, whey cheese, processed cheese, cheese analogues, whey protein cheese that can be classified based on physical characteristics as hard (e.g. Parmesan), semi-hard (e.g. cheddar), medium-hard (e.g. edam), semi-soft and soft (e.g. mozzarella, paneer, cottage, ricotta, cream cheese) as well as serving style as slice, grated or spreadable cheese |  |
| Mixed dishes | 9 (a) | Western-style prepared food | Calorie-dense and nutrient-poor foods that contains high levels of calories, total fat, saturated fat, sugar, or sodium (e.g. pizza, lasagna, burgers, soups, French fries, onion rings, coleslaw, crumbed and/or battered chicken), sandwiches prepared upon order | Uncooked meats (Category 13 or 14) |
| Mixed dishes | 9 (b) | Asian-style prepared food | Food that typically comes with a carbohydrate staple such as rice, noodles, Indian breads (e.g. prata, naan, chapati), Indian pancakes and similar. Includes hawker food such as hokkien mee, char kuey teow, Chinese stir fry, nasi lemak, etc. |  |
| Mixed dishes | 9 (c) | Instant & Convenience Packed Food | Food that requires minimal preparation/ cooking before consumption. , creamed corn soup, luncheon meat, tinned fruits), ready-to-eat food (bentos, ready-made sandwiches), instant food (e.g. tinned spaghetti, instant noodles, instant porridge) |  |
| Non-core food | 10 | Butter and other fats and oils | Butter, vegetable oils, margarine and spreads, lard, dripping, ghee |  |
| Core food | 11 | Bread, bread products and crisp breads | Bread (i.e. white, yellow, whole wheat) and rolls, pita, naan, rotis, prata, wraps/tortillas, bread with fillings (e.g. raisins bread, kaya butter toast, cheese bread), buttered toasted bread, steamed bread and buns, mixes for making bread |  |
| Core food | 12 | Noodles, pasta, rice, and grains | Fresh, precooked, or dried noodles, vermicelli (e.g. made from wheat, tapioca, sago, legume, etc.), pasta (e.g. cooked with sauce, agio olio), grains (e.g. quinoa), rice (e.g. red, brown, white, basmati, porridge). Includes both cooked and uncooked. | Instant noodles, instant porridge (Category 9c) |
| Core food | 13 | Fresh and frozen meat, poultry, fish, and seafood products | Fresh and frozen meat (e.g. beef, pork, chicken, lamb, goat, duck), eggs, seafood (e.g. prawns, sea urchin, herring, tuna, mackerel) |  |
| Non-core food | 14 | Processed meat, poultry, fish, and seafood products | Non-heat and heat treated, comminuted meat poultry that have been cured/ cured and dried or fermented. E.g. sausage, ham, bacon, pepperoni, salami, chorizo, fish balls, fish/chicken nuggets, fish fingers, processed beef or chicken patty, canned meat (e.g. luncheon) |  |
| Core food | 15 | Fruit, vegetable, legumes, fungus | Potatoes, roots crops, fresh coconut, mushrooms, dried fruit, dried coconut, vegetables, and legumes (e.g. soya beans, peanuts), dried mushrooms, preserved or pickled fruits and vegetables, fermented vegetables | 100% fruit and vegetable juices (Category 4a), sugar sweetened fruit juices (Category 4d), canned fruits (Category 9c) |
| Core food | 16 | Products made from soya | Tofu products, natto, tempeh | Soya sauce (Category 18), oils from soya (Category 10), fresh soya beans (Category 15) |
| Non-core food | 17 | Sauces, dips, and dressings | Tomato ketchup, coloured ketchup, mayonnaise, salad dressing, soya sauce, fish sauce, sweet chili sauce, gravies, spaghetti sauce, barbecue sauces, seasonings, reconstituted stocks |  |
| Core food | 18 | Health supplements, traditional Chinese herbs, and tonics | Vitamins, supplements (pills, powder, drinks), Bird's Nest, Ginseng extract, Chicken Essence, spices (e.g. Cinnamon, star anise) |  |

Supplementary Table 3 Marketing techniques present on Facebook and Instagram accounts of top 5 beverage, FMCG, and retail food brands in Singapore

| Marketing Techniques | 100PLUS,  n (%) | Coca-Cola,  n (%) | LiHO,  n (%) | MILO,  n (%) | Nescafe,  n (%) | Ben & Jerry's,  n (%) | Ferrero Rocher,  n (%) | Kinder Bueno,  n (%) | KitKat,  n (%) | Sticky,  n (%) | Foodpanda,  n (%) | KFC,  n (%) | McDonald's,  n (%) | Starbucks,  n (%) | Subway,  n (%) |
| --- | --- | --- | --- | --- | --- | --- | --- | --- | --- | --- | --- | --- | --- | --- | --- |
| **Branding subtotal** | **74 (100)** | **61 (100)** | **176 (100)** | **28**  **(100)** | **6**  **(100)** | **63**  **(100)** | **26**  **(100)** | **24**  **(100)** | **22**  **(100)** | **61 (100)** | **236 (100)** | **123 (100)** | **131**  **(100)** | **133**  **(100)** | **97**  **(100)** |
| Branding elements | 74 (100) | 61 (100) | 173  (98) | 28  (100) | 6  (100) | 55  (87) | 26  (100) | 24  (100) | 21  (95) | 61 (100) | 192  (81) | 120  (98) | 116  (89) | 120  (90) | 97  (100) |
| Branded Characters | 12  (16) | 0  (0) | 143  (81) | 0  (0) | 0  (0) | 32  (51) | 7  (27) | 7  (29) | 5  (23) | 16  (26) | 166  (70) | 34  (28) | 7  (5) | 90  (68) | 14  (14) |
| **Characters subtotal** | **39 (53)** | **36 (59)** | **45 (26)** | **17**  **(61)** | **1**  **(17)** | **0**  **(0)** | **0**  **(0)** | **0**  **(0)** | **1**  **(5)** | **4**  **(7)** | **3**  **(1)** | **12 (10)** | **7**  **(5)** | **3**  **(2)** | **0**  **(0)** |
| Celebrities and/or sportsperson | 20  (27) | 0  (0) | 21  (12) | 11  (39) | 0  (0) | 0  (0) | 0  (0) | 0  (0) | 0  (0) | 0  (0) | 1  (0) | 2  (2) | 1  (1) | 0  (0) | 0  (0) |
| Other characters | 10  (14) | 8  (13) | 16  (9) | 9  (32) | 1  (17) | 0  (0) | 0  (0) | 0  (0) | 1  (5) | 2  (3) | 3  (1) | 10  (8) | 12  (9) | 4  (3) | 0  (0) |
| **Events and/or festivals** | **29**  **(39)** | **46**  **(75)** | **86**  **(49)** | **10**  **(36)** | **0**  **(0)** | **23**  **(37)** | **20**  **(77)** | **2**  **(8)** | **8**  **(36)** | **27**  **(44)** | **42**  **(18)** | **35**  **(28)** | **61**  **(47)** | **15**  **(11)** | **10**  **(10)** |
| **Premium offer subtotal** | **26 (35)** | **42 (69)** | **70 (40)** | **20**  **(71)** | **3**  **(50)** | **29**  **(46)** | **0**  **(0)** | **1**  **(4)** | **17 (77)** | **7**  **(11)** | **143 (61)** | **91 (74)** | **93**  **(71)** | **97**  **(73)** | **32**  **(33)** |
| Contests and/or giveaways | 18  (24) | 21  (34) | 31  (18) | 20  (71) | 3  (50) | 18  (29) | 0  (0) | 0  (0) | 3  (14) | 0  (0) | 24  (10) | 16  (13) | 5  (4) | 6  (5) | 2  (2) |
| Special price promotions | 0  (0) | 0  (0) | 6  (3) | 0  (0) | 0  (0) | 8  (13) | 0  (0) | 0  (0) | 5  (23) | 2  (3) | 114  (48) | 22  (18) | 9  (7) | 11  (8) | 16  (16) |
| Limited edition merchandises and/or seasonal products | 12  (16) | 33  (54) | 30  (17) | 2  (7) | 0  (0) | 4  (6) | 0  (0) | 1 (4) | 9  (41) | 7  (11) | 18  (8) | 71  (58) | 71  (54) | 63  (47) | 27  (28) |
| Gift or collectables | 6  (8) | 29  (48) | 0  (0) | 5  (18) | 0  (0) | 2  (3) | 0  (0) | 0  (0) | 3  (14) | 0  (0) | 12  (5) | 4  (3) | 3 (2) | 29  (22) | 2  (2) |
| Exclusive offers for people who like and/or comment on | 0  (0) | 0  (0) | 8  (5) | 0  (0) | 0  (0) | 1  (2) | 0  (0) | 0  (0) | 0  (0) | 0  (0) | 5  (2) | 8  (7) | 4  (3) | 8  (6) | 0  (0) |
| Membership or loyalty rewards | 0  (0) | 0  (0) | 0  (0) | 0  (0) | 0  (0) | 0  (0) | 0  (0) | 0  (0) | 0  (0) | 0  (0) | 9 (4) | 0  (0) | 0  (0) | 7  (5) | 0  (0) |
| Game and app downloads | 0  (0) | 2  (3) | 0  (0) | 0  (0) | 0  (0) | 0  (0) | 0  (0) | 0  (0) | 0  (0) | 0  (0) | 4  (2) | 3  (2) | 25  (19) | 5  (4) | 0  (0) |
| Free extra ingredients | 0  (0) | 0  (0) | 0  (0) | 3  (11) | 0  (0) | 0  (0) | 0  (0) | 0  (0) | 4  (18) | 0  (0) | 0  (0) | 0  (0) | 0  (0) | 0  (0) | 0  (0) |
| **Health claims description subtotal** | **50**  **(68)** | **20 (33)** | **14**  **(8)** | **24**  **(86)** | **1**  **(17)** | **2**  **(3)** | **0**  **(0)** | **0**  **(0)** | **0**  **(0)** | **0**  **(0)** | **9**  **(4)** | **5**  **(4)** | **8**  **(6)** | **2**  **(2)** | **15**  **(15)** |
| Health related ingredients claims | 0  (0) | 16  (26) | 5  (3) | 5  (18) | 0  (0) | 1  (2) | 0  (0) | 0  (0) | 0  (0) | 0  (0) | 3  (1) | 0  (0) | 4  (3) | 2  (2) | 2  (2) |
| Nutrient related claims | 38  (51) | 8  (13) | 7  (4) | 17  (61) | 1  (17) | 0  (0) | 0  (0) | 0  (0) | 0  (0) | 0  (0) | 7  (3) | 2  (2) | 0  (0) | 0  (0) | 11  (11) |
| Health claims | 2  (3) | 0  (0) | 7  (4) | 9  (32) | 0  (0) | 0  (0) | 0  (0) | 0  (0) | 0  (0) | 0  (0) | 4  (2) | 1  (1) | 3  (2) | 1  (1) | 4  (4) |
| Healthier Choice Symbol | 38  (51) | 13  (21) | 0  (0) | 18  (64) | 0  (0) | 0  (0) | 0  (0) | 0  (0) | 0  (0) | 0  (0) | 3  (1) | 4  (3) | 2  (2) | 0  (0) | 4  (4) |
| Other health claims | 3  (4) | 0  (0) | 6  (3) | 7  (25) | 0  (0) | 1  (2) | 0  (0) | 0  (0) | 0  (0) | 0  (0) | 1  (0) | 0  (0) | 1  (1) | 0  (0) | 0  (0) |
| **Brand benefit claims subtotal** | **52 (70)** | **46 (75)** | **145 (82)** | **24**  **(86)** | **5**  **(83)** | **60**  **(95)** | **9**  **(35)** | **20**  **(83)** | **21 (95)** | **57 (93)** | **219 (93)** | **116 (94)** | **120**  **(92)** | **110**  **(83)** | **71**  **(73)** |
| Sensory based characteristics | 15  (20) | 28  (46) | 110  (63) | 13  (46) | 4  (67) | 57  (90) | 1  (4) | 18  (75) | 17  (77) | 51  (84) | 214  (91) | 107  (87) | 101  (77) | 76  (57) | 64  (66) |
| New brand development | 1  (1) | 13  (21) | 45  (26) | 10  (36) | 3  (50) | 3  (5) | 0  (0) | 0  (0) | 4  (18) | 0  (0) | 5  (2) | 39  (32) | 40  (31) | 24  (18) | 8  (8) |
| Suggested use | 45  (61) | 32  (52) | 63  (36) | 14  (50) | 4  (67) | 9  (14) | 8  (31) | 11  (46) | 17  (77) | 33  (54) | 44  (19) | 61  (50) | 46  (35) | 50  (38) | 39  (40) |
| Suggested users are children or whole family | 2  (3) | 0  (0) | 19  (11) | 5  (18) | 0  (0) | 0  (0) | 0  (0) | 0  (0) | 2  (9) | 0  (0) | 1  (0) | 3  (2) | 6  (5) | 0  (0) | 0  (0) |
| Emotive claims | 18  (24) | 21  (34) | 60  (34) | 9  (32) | 5  (83) | 4  (6) | 1  (4) | 3  (13) | 6  (27) | 14  (23) | 21  (9) | 36  (29) | 66  (50) | 30  (23) | 19  (20) |
| Puffery | 3  (4) | 0  (0) | 2  (1) | 1  (4) | 0  (0) | 11  (17) | 0  (0) | 0  (0) | 1  (5) | 4  (7) | 2  (1) | 7  (6) | 7  (5) | 9  (7) | 3  (3) |
| Convenience | 1  (1) | 0  (0) | 2  (1) | 1  (4) | 1  (17) | 1  (2) | 0  (0) | 0  (0) | 4  (18) | 4  (7) | 94  (40) | 25  (20) | 43  (33) | 1  (1) | 9  (9) |
| **CSR^c^ and/or philanthropy subtotal** | **0**  **(0)** | **0**  **(0)** | **2**  **(1)** | **0**  **(0)** | **0**  **(0)** | **25**  **(40)** | **0**  **(0)** | **0**  **(0)** | **0**  **(0)** | **0**  **(0)** | **4**  **(2)** | **3**  **(2)** | **2**  **(2)** | **1**  **(1)** | **0**  **(0)** |
| CSR^c^ | 0  (0) | 0  (0) | 2  (1) | 0  (0) | 0  (0) | 25  (40) | 0  (0) | 0  (0) | 0  (0) | 0  (0) | 4  (2) | 2  (2) | 2  (2) | 1  (1) | 0  (0) |
| Philanthropy | 0  (0) | 0  (0) | 2  (1) | 0  (0) | 0  (0) | 2  (3) | 0  (0) | 0  (0) | 0  (0) | 0  (0) | 0  (0) | 1  (1) | 0  (0) | 0  (0) | 0  (0) |
| **Engagement subtotal** | **24 (32)** | **34 (56)** | **66 (38)** | **22**  **(79)** | **3**  **(50)** | **41**  **(65)** | **9**  **(35)** | **12**  **(50)** | **3**  **(14)** | **7**  **(11)** | **165 (70)** | **74 (60)** | **68**  **(52)** | **39**  **(29)** | **59**  **(61)** |
| Activities, games & quizzes | 6  (8) | 4  (7) | 20  (11) | 3  (11) | 3  (50) | 13  (21) | 9  (35) | 11  (46) | 0  (0) | 2  (3) | 12  (5) | 13  (11) | 12  (9) | 18  (14) | 28  (29) |
| Polls | 0  (0) | 0  (0) | 2  (1) | 0  (0) | 0  (0) | 0  (0) | 0  (0) | 0  (0) | 0  (0) | 0  (0) | 34  (14) | 5  (4) | 1  (1) | 1  (1) | 13  (13) |
| Conversations | 12  (16) | 20  (33) | 45  (26) | 21  (75) | 2  (33) | 35  (56) | 0  (0) | 0  (0) | 2  (9) | 5  (8) | 146  (62) | 65  (53) | 53  (40) | 20  (15) | 33  (34) |
| Advergaming | 0  (0) | 0  (0) | 0  (0) | 0  (0) | 0  (0) | 0  (0) | 0  (0) | 0  (0) | 1  (5) | 0  (0) | 0  (0) | 0  (0) | 1  (1) | 0  (0) | 0  (0) |
| Storyline | 6  (8) | 9  (15) | 0  (0) | 0  (0) | 0  (0) | 0  (0) | 0  (0) | 1  (4) | 0  (0) | 0  (0) | 0  (0) | 1  (1) | 4  (3) | 0  (0) | 0  (0) |
| Community engagement/ challenge | 6  (8) | 8  (13) | 11  (6) | 1  (4) | 0  (0) | 0  (0) | 0  (0) | 0  (0) | 0  (0) | 0  (0) | 1  (0) | 1  (1) | 1  (1) | 2  (2) | 0  (0) |
| **Viral marketing subtotal** | **58 (78)** | **48 (79)** | **164 (93)** | **8**  **(29)** | **2**  **(33)** | **37**  **(59)** | **4**  **(15)** | **22**  **(92)** | **2**  **(9)** | **59 (97)** | **6**  **(3)** | **106 (86)** | **28**  **(21)** | **59**  **(44)** | **18**  **(19)** |
| Tag | 1  (1) | 2  (3) | 18  (10) | 2  (7) | 1  (17) | 0  (0) | 0  (0) | 4  (17) | 0  (0) | 0  (0) | 0  (0) | 8  (7) | 1  (1) | 2  (2) | 6  (6) |
| Share / post | 1  (1) | 0  (0) | 7  (4) | 5  (18) | 0  (0) | 0  (0) | 0  (0) | 1  (4) | 0  (0) | 0  (0) | 1  (0) | 0  (0) | 2  (2) | 2  (2) | 0  (0) |
| Hashtag | 58  (78) | 48  (79) | 162  (92) | 8  (29) | 1  (17) | 37  (59) | 4  (15) | 22  (92) | 2  (9) | 59  (97) | 4  (2) | 103  (84) | 27  (21) | 57  (43) | 12  (12) |
| Like | 1  (1) | 0  (0) | 9  (5) | 0  (0) | 0  (0) | 0  (0) | 0  (0) | 0  (0) | 0  (0) | 0  (0) | 3  (1) | 1  (1) | 0  (0) | 0  (0) | 0  (0) |
| Follow | 0  (0) | 2  (3) | 4  (2) | 2  (7) | 0  (0) | 0  (0) | 0  (0) | 0  (0) | 0  (0) | 2  (3) | 2  (1) | 0  (0) | 0  (0) | 0  (0) | 0  (0) |
| **Sponsorships and/or partnerships subtotal** | **25 (34)** | **24 (39)** | **33 (19)** | **20**  **(71)** | **0**  **(0)** | **3**  **(5)** | **0**  **(0)** | **0**  **(0)** | **2**  **(9)** | **0**  **(0)** | **142 (60)** | **10**  **(8)** | **27**  **(21)** | **14**  **(11)** | **9**  **(9)** |
| Sponsorships | 16  (22) | 1  (2) | 12  (7) | 9  (32) | 0  (0) | 1  (2) | 0  (0) | 0  (0) | 0  (0) | 0  (0) | 3  (1) | 5  (4) | 0  (0) | 0  (0) | 1  (1) |
| Partnerships | 17  (23) | 23  (38) | 21  (12) | 12  (43) | 0  (0) | 2  (3) | 0  (0) | 0  (0) | 2  (9) | 0  (0) | 139  (59) | 6  (5) | 27  (21) | 14  (11) | 8  (8) |
| **Links and multimedia subtotal** | **45 (61)** | **49 (80)** | **111 (63)** | **22 (79)** | **6 (100)** | **43 (68)** | **26**  **(100)** | **24 (100)** | **22 (100)** | **32 (52)** | **199 (84)** | **82 (67)** | **94**  **(72)** | **79**  **(59)** | **92**  **(95)** |
| Links | 37  (50 | 31  (51) | 102  (58) | 17  (61) | 5  (83) | 30  (48) | 6  (23) | 0  (0) | 3  (14) | 32  (52) | 171  (72) | 64  (52) | 55  (42) | 51  (38) | 24  (25) |
| GIF and/or Boomerang | 4  (5) | 0  (0) | 6  (3) | 0  (0) | 0  (0) | 1  (2) | 0  (0) | 0  (0) | 0  (0) | 0  (0) | 5  (2) | 4  (3) | 3  (2) | 16  (12) | 5  (5) |
| Photos | 42  (57) | 29  (48) | 162  (92) | 14  (50) | 3  (50) | 35  (56) | 16  (62) | 23  (96) | 6  (27) | 61  (100) | 197  (83) | 71  (58) | 49  (37) | 92  (69) | 81  (84) |
| Videos | 29  (39) | 33  (54) | 8  (5) | 13  (46) | 3  (50) | 27  (43) | 10  (38) | 1  (4) | 16  (73) | 0  (0) | 32  (14) | 49  (40) | 77  (59) | 26  (20) | 4  (4) |
| **Advercation (merged)** | **4**  **(5)** | **1**  **(2)** | **11**  **(6)** | **1**  **(4)** | **0**  **(0)** | **3**  **(5)** | **0**  **(0)** | **4**  **(17)** | **1**  **(5)** | **0**  **(0)** | **30**  **(13)** | **9**  **(7)** | **8**  **(6)** | **3**  **(2)** | **5**  **(5)** |
| **UGC^d^** | **5**  **(7)** | **0**  **(0)** | **6**  **(3)** | **1**  **(4)** | **0**  **(0)** | **3**  **(5)** | **0**  **(0)** | **0**  **(0)** | **0**  **(0)** | **8**  **(13)** | **0**  **(0)** | **2**  **(2)** | **0**  **(0)** | **0**  **(0)** | **0**  **(0)** |

^a^ Website communities/forums was not observed in any posts.

^b^ Column percentages that add up to more than 100% are where multiple marketing techniques were coded in a single post.

^c^ Corporate social responsibility

^d^ User generated content

Supplementary Table 4 Food referenced on Facebook and Instagram by top 5 beverage, FMCG, and retail food brands in Singapore

| Food category | 100PLUS,  n (%) | Coca-Cola,  n (%) | LiHO,  n (%) | MILO,  n (%) | Nescafe,  n (%) | Ben & Jerry's,  n (%) | Ferrero Rocher,  n (%) | Kinder Bueno,  n (%) | KitKat,  n (%) | Sticky,  n (%) | Foodpanda,  n (%) | KFC,  n (%) | McDonald's,  n (%) | Starbucks,  n (%) | Subway,  n (%) |
| --- | --- | --- | --- | --- | --- | --- | --- | --- | --- | --- | --- | --- | --- | --- | --- |
| **Core food subtotal** | **9 (12)** | **4 (7)** | **57 (32)** | **10 (36)** | **0 (0)** | **3 (5)** | **0 (0)** | **0 (0)** | **2 (9)** | **0 (0)** | **71 (30)** | **19 (15)** | **30 (23)** | **13 (10)** | **11 (11)** |
| Milk, yoghurt, cream, and cheese | 1 (1) | 0 (0) | 15 (9) | 1 (4) | 0 (0) | 0 (0) | 0 (0) | 0 (0) | 1 (5) | 0 (0) | 4 (2) | 3 (2) | 3 (2) | 3 (2) | 0 (0) |
| Breakfast cereals | 0 (0) | 0 (0) | 0 (0) | 0 (0) | 0 (0) | 1 (2) | 0 (0) | 0 (0) | 0 (0) | 0 (0) | 2 (1) | 0 (0) | 1 (1) | 2 (2) | 0 (0) |
| Water and/or non-sweetened beverages | 0 (0) | 0 (0) | 4 (2) | 0 (0) | 0 (0) | 0 (0) | 0 (0 | 0 (0) | 0 (0) | 0 (0) | 0 (0) | 1 (1) | 2 (2) | 1 (1) | 0 (0) |
| Bread, bread products and crisp breads | 0 (0) | 0 (0) | 0 (0) | 2 (7) | 0 (0) | 0 (0) | 0 (0) | 0 (0) | 0 (0) | 0 (0) | 5 (2) | 2 (2) | 3 (2) | 1 (1) | 2 (2) |
| Noodles, pasta, rice, and grains | 0 (0) | 1 (2) | 0 (0) | 2 (7) | 0 (0) | 0 (0) | 0 (0) | 0 (0) | 0 (0) | 0 (0) | 0 (0) | 4 (3) | 0 (0) | 0 (0) | 0 (0) |
| Fresh meat, poultry, fish | 0 (0) | 0 (0) | 0 (0) | 0 (0) | 0 (0) | 0 (0) | 0 (0) | 0 (0) | 0 (0) | 0 (0) | 4 (2) | 1 (1) | 9 (7) | 0 (0) | 0 (0) |
| Fruit, vegetables, legumes | 8 (11) | 3 (5) | 38 (22) | 5 (18) | 0 (0) | 2 (3) | 0 (0) | 0 (0) | 1 (5) | 0 (0) | 52 (22) | 8 (7) | 12 (9) | 6 (5) | 9 (9) |
| **Non-core food subtotal** | **74 (100)** | **48 (79)** | **165 (94)** | **25 (89)** | **5 (83)** | **73 (116)** | **22 (85)** | **18 (75)** | **19 (86)** | **57 (93)** | **124 (53)** | **72 (59)** | **113 (86)** | **114 (86)** | **55 (57)** |
| Sweet snacks | 6 (8) | 0 (0) | 21 (12) | 3 (11) | 0 (0) | 17 (27) | 20 (77) | 17 (71) | 18 (82) | 57 (93) | 25 (11) | 8 (7) | 22 (17) | 13 (10) | 28 (29) |
| Savoury snacks | 5 (7) | 0 (0) | 2 (1) | 0 (0) | 0 (0) | 1 (2) | 0 (0) | 1 (4) | 0 (0) | 0 (0) | 8 (3) | 4 (3) | 0 (0) | 1 (1) | 1 (1) |
| Tea and coffee | 0 (0) | 0 (0) | 11 (6) | 0 (0) | 5 (83) | 0 (0) | 1 (4) | 0 (0) | 0 (0) | 0 (0) | 2 (1) | 4 (3) | 18 (14) | 80 (60) | 5 (5) |
| Sweetened beverages | 59 (80) | (79) | 129 (73) | 22 (79) | 0 (0) | 3 (5) | 0 (0) | 0 (0) | 0 (0) | 0 (0) | 24 (10) | 22 (18) | 30 (23) | 17 (13) | 16 (16) |
| Alcoholic beverages | 0 (0) | 0 (0) | 0 (0) | 0 (0) | 0 (0) | 0 (0) | 0 (0) | 0 (0) | 0 (0) | 0 (0) | 4 (2) | 0 (0) | 0 (0) | 0 (0) | 0 (0) |
| Edible ices | 1 (1) | 0 (0) | 0 (0) | 0 (0) | 0 (0) | 52 (83) | 0 (0) | 0 (0) | 1 (5) | 0 (0) | 12 (5) | 4 (3) | 13 (10) | 0 (0) | 0 (0) |
| Butter and other fats and oils | 0 (0) | 0 (0) | 0 (0) | 0 (0) | 0 (0) | 0 (0) | 0 (0) | 0 (0) | 0 (0) | 0 (0) | 1 (0) | 0 (0) | 0 (0) | 1 (1) | 0 (0) |
| Processed meat, poultry, and fish | 1 (1) | 0 (0) | 0 (0) | 0 (0) | 0 (0) | 0 (0) | 1 (4) | 0 (0) | 0 (0) | 0 (0) | 1 (0) | 0 (0) | 10 (8) | 0 (0) | 1 (1) |
| Sauces, dips, and dressings | 2 (3) | 0 (0) | 2 (1) | 0 (0) | 0 (0) | 0 (0) | 0 (0) | 0 (0) | 0 (0) | 0 (0) | 47 (20) | 30 (24) | 20 (15) | 2 (2) | 4 (4) |
| **Mixed dishes subtotal** | **8 (11)** | **4 (7)** | **7 (4)** | **3 (11)** | **1 (17)** | **0 (0)** | **0 (0)** | **0 (0)** | **0 (0)** | **0 (0)** | **192 (81)** | **110 (89)** | **92 (70)** | **14 (11)** | **55 (57)** |
| Asian styled prepared food | 2 (3) | 2 (3) | 5 (3) | 0 (0) | 1 (17) | 0 (0) | 0 (0) | 0 (0) | 0 (0) | 0 (0) | 105 (44) | 6 (5) | 0 (0) | 2 (2) | 0 (0) |
| Western styled prepared food | 6 (8) | 2 (3) | 2 (1) | 3 (11) | 0 (0) | 0 (0) | 0 (0) | 0 (0) | 0 (0) | 0 (0) | 87 (37) | 104 (85) | 92 (70) | 12 (9) | 55 (57) |

^a^ Percentages that add up to more than 100% are where multiple foods were coded in a single post

^b^ The following food groups (Soya products, Supplements, herbs, and tonics, Instant and convenience packed food) were not observed in any posts.
